# Applications of Anti-Cytomegalovirus T Cells for Cancer (Immuno)Therapy

**DOI:** 10.3390/cancers15153767

**Published:** 2023-07-25

**Authors:** Isabel Britsch, Anne Paulien van Wijngaarden, Wijnand Helfrich

**Affiliations:** Department of Surgery, Translational Surgical Oncology, University of Groningen, UMC Groningen, Hanzeplein 1, 9713 GZ Groningen, The Netherlands; i.britsch@umcg.nl (I.B.);

**Keywords:** cancer immunotherapy, CMV, T cells, memory inflation, ACT

## Abstract

**Simple Summary:**

Numerous studies have developed strategies to utilize anti-cytomegalovirus (CMV) T cells for cancer treatment, as they have many beneficial characteristics including extraordinarily high numbers and frequent presence in cancer tissues. In this review, we present multiple strategies that exploit anti-CMV T cells for cancer (immuno)therapy in various ways. We aim to advance the understanding of how anti-CMV T cells can be applied best to further improve treatment outcomes for cancer patients. For this purpose, we identify similarities and discuss benefits, disadvantages, and challenges of each strategy. Finally, we comment on the future directions of this new promising field of cancer (immuno)therapy.

**Abstract:**

Infection with cytomegalovirus (CMV) is highly prevalent in the general population and largely controlled by CD8^pos^ T cells. Intriguingly, anti-CMV T cells accumulate over time to extraordinarily high numbers, are frequently present as tumor-resident ‘bystander’ T cells, and remain functional in cancer patients. Consequently, various strategies for redirecting anti-CMV CD8^pos^ T cells to eliminate cancer cells are currently being developed. Here, we provide an overview of these strategies including immunogenic CMV peptide-loading onto endogenous HLA complexes on cancer cells and the use of tumor-directed fusion proteins containing a preassembled CMV peptide/HLA-I complex. Additionally, we discuss conveying the advantageous characteristics of anti-CMV T cells in adoptive cell therapy. Utilization of anti-CMV CD8^pos^ T cells to generate CAR T cells promotes their in vivo persistence and expansion due to appropriate co-stimulation through the endogenous (CMV-)TCR signaling complex. Designing TCR-engineered T cells is more challenging, as the artificial and endogenous TCR compete for expression. Moreover, the use of expanded/reactivated anti-CMV T cells to target CMV peptide-expressing glioblastomas is discussed. This review highlights the most important findings and compares the benefits, disadvantages, and challenges of each strategy. Finally, we discuss how anti-CMV T cell therapies can be further improved to enhance treatment efficacy.

## 1. Introduction

The cytomegalovirus (CMV) is a herpesvirus with an estimated global seroprevalence of 83% [1]. However, infection rates vary significantly depending on demographic and socioeconomic factors [2,3,4]. Typically, CMV infection occurs during childhood, is asymptomatic in healthy individuals, and leads to lifelong presence in the host. CMV infection is largely controlled by anti-CMV CD8^pos^ T cell clones. Notably, an extensive number of studies have observed exceptionally high frequencies of anti-CMV CD8^pos^ T cells in (primarily elderly) individuals [5,6,7,8,9].

In clinical practice, treatment with anti-CMV T cells is mainly used to prevent CMV disease after hematopoietic stem cell transplantation. Currently, an increasing number of studies are investigating alternative approaches for utilizing anti-CMV T cells in cancer (immuno)therapy. Here, we discuss various strategies to exploit anti-CMV T cells for the treatment of solid and non-solid malignancies.

## 2. Anti-CMV T Cells—Potent Effector Cells for Cancer Treatment

Researchers have provided compelling lines of evidence that anti-CMV T cells may be particularly suitable for use in cancer (immuno)therapy. Firstly, CMV infection is controlled lifelong in both healthy individuals and cancer patients. The latter indicates that anti-CMV T cell responses remain functional and potent in cancer patients. Indeed, anti-CMV T cell responses appear to be intact even in cancer patients with malignancies such as chronic lymphocytic leukemia (CLL), in which the majority of CD8^pos^ T cells are anergic [10]. Secondly, there is evidence that in various cancer types, anti-CMV T cells are abundantly present as tumor-resident ‘bystander’ T cells. In particular, functional anti-CMV T cells have been found to populate solid tumors in colon and lung [11,12]. Thirdly, the anti-CMV T cell population may become extraordinarily large over time. In healthy CMV-seropositive individuals, up to 9% and 10% of circulating T cells may account for anti-CMV CD4^pos^ and CD8^pos^ T cells, respectively. Remarkably, in CMV-seropositive cancer patients, the frequency of anti-CMV CD8^pos^ T cells can be even higher than in healthy individuals, reaching up to 20% [5]. These extraordinarily high numbers of anti-CMV T cells are due to a phenomenon known as ‘memory T cell inflation’, as discussed below.

## 3. Memory T Cell Inflation

T cell responses to CMV are driven by the lifelong presence of the virus in the host. Clearance of infected and virus-producing cells resolves the primary infection, but in some cells viral genomes remain in a quiescent state. In particular, bone marrow-resident CD34^pos^ hematopoietic stem and progenitor cells are a critical reservoir of latent human CMV (HCMV) infection, which is maintained during differentiation into CD14^pos^ monocytes [13,14,15]. Viral reactivation occurs upon further cellular differentiation and/or under pro-inflammatory conditions [16,17]. Repetitively occurring reactivation over time ensures that the levels of anti-CMV T cells do not contract but are rather maintained and accumulate at high frequencies. This phenomenon, known as memory T cell inflation, was first described for murine CMV (MCMV) [18] and has been confirmed in numerous studies [19,20,21,22]. Indications for memory inflation occurring in HCMV have been observed in several cross-sectional studies investigating CMV-seropositive elderly individuals [5,7,8,23,24].

Although inflationary anti-CMV CD8^pos^ T cells are repeatedly exposed to CMV antigens, they remain polyfunctional [23,25,26] and do not show signs of exhaustion in mice [27] or humans [28]. In particular, anti-CMV CD8^pos^ T cells expose low levels of inhibitory markers such as PD-1 and retain their capacity to proliferate, secrete cytokines, and induce cytotoxicity [29]. Mouse and human inflationary anti-CMV CD8^pos^ T cells have an effector–memory phenotype (CD27^low^, CD28^neg^, CD127^neg^, and IL-2^pos/neg^) and accumulate in non-lymphoid peripheral organs [20,21,30,31]. In line with their phenotype, inflationary anti-CMV CD8^pos^ T cells maintain a functional ‘ready-to-go state’ by exploiting alternative, rather than classical, co-stimulatory signals [32,33]. Moreover, inflationary anti-CMV CD8^pos^ T cells express the chemokine receptors CX3CR1 and CXCR3, which enables them to infiltrate inflamed tissues [29,34]. In humans, the antigen response of anti-HCMV CD8^pos^ T cells is highly diverse, and responses to the immunodominant viral proteins pp65 and IE-1 are the best studied [35,36].

Notably, memory T cell inflation is not exclusive to CMV, as it is also observed for other latent virus types such as herpes simplex virus (HSV)-1 and Epstein–Barr virus (EBV) [37].

## 4. Strategies to Exploit Inflationary Anti-CMV T Cells for Elimination of Cancer Cells

Due to the above-mentioned beneficial characteristics of anti-CMV CD8^pos^ T cells, such as their abundance and constant renewability, various strategies have been developed to exploit these potent cytolytic effector cells for cancer immunotherapy. In contrast to naïve T cells, antigen-experienced inflationary anti-CMV CD8^pos^ T cells do not require classical priming through presentation of peptides by dendritic cells or other professional antigen-presenting cells [38].

The strategies described in this section all aim to mimic CMV infection by (re)decorating malignant cells with viral antigens on (endogenous or exogenous) human leukocyte antigen class I (HLA-I) complexes. In this way, anti-CMV CD8^pos^ T cells can recognize and eliminate cancer cells.

### 4.1. Loading CMV Peptides into Endogenous HLA Complexes on Cancer Cells

The strategies described in the following paragraphs aim to load CMV peptides into endogenous HLA complexes on cancer cells to render these cells recognizable by anti-CMV CD8^pos^ T cells. Among the examples are the so-called T cell epitope-delivering antibodies (TEDbodies), which can be used to selectively deliver CMV peptides into the cytosol of cancer cells. Once in the cytosol, the CMV peptide moiety of the TEDbody enters the natural antigen-processing and -presentation machinery of the targeted cancer cells and is ultimately presented by endogenous HLA complexes. In contrast, so-called antibody–peptide epitope conjugates (APECs) do not enter the cytosol but are externally loaded into ‘empty’ HLA-I molecules on the surface of cancer cells. Moreover, anti-CMV T cell responses can be activated locally by intratumoral (i.t.) injection of CMV peptide epitopes, which are also externally loaded into cell surface-expressed ‘empty’ HLA-I (and HLA-II).

#### 4.1.1. TEDbodies

**Figure 1:**
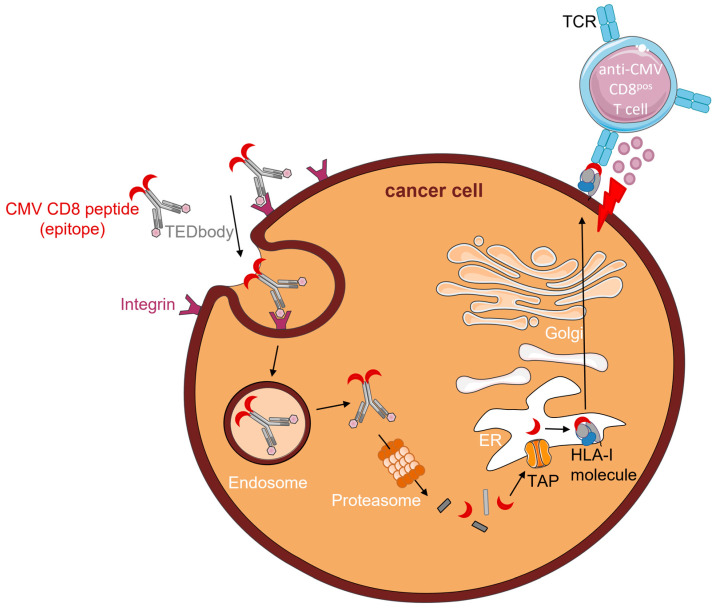
Proposed mode of action of TEDbodies (adapted from Jung et al. [39]). TEDbodies deliver HLA-I-restricted CMV peptide epitopes to cancer cells, thereby rendering them susceptible to elimination by pre-existing (inflationary) anti-CMV CD8^pos^ T cells. TEDbodies bind to integrin αvβ5/αvβ3 on the surface of cancer cells, and are internalized and then relocated into the cytosol through endosomal escape. Subsequent cleavage by proteasomes creates precursor CMV peptides that are taken up into the ER and N-terminally trimmed. Mature CMV peptides bind to the cognate HLA-I complex and are transported through the ER–Golgi pathway to the surface of cancer cells. Anti-CMV CD8^pos^ T cells then recognize and eliminate CMV peptide-presenting cancer cells.

Recently, Jung et al. (2022) described CD8^pos^ T cell epitope-delivering antibodies (TEDbodies) [39]. TEDbodies consist of a full-length human IgG1/κ antibody with an effector function-silenced Fc domain. The VL and VH domains are designed to facilitate endosomal escape of TEDbodies into the cytosol. The CMV protein pp65-derived HLA-A*02:01 peptide epitope NLV (or an N/C-extended version thereof) was fused to the Fc domain of the antibody. Integrin αvβ5/αvβ3-targeting cyclic peptide ‘in4′ was fused to the light chain N-terminus, which facilitates tissue-homing and receptor-mediated endocytosis of TEDbodies. Of note, integrin αvβ5/αvβ3 is overexpressed in many types of epithelial cancers. Upon binding to integrin αvβ5/αvβ3 on the cancer cell surface, TEDbodies are endocytosed. Owing to the endosomal escape activity of their VL and VH domains, TEDbodies end up in the cytoplasm, where they are cleaved by cytosolic proteasomes, creating precursor CMV peptides with the correct C-terminus. These peptides are then transported by transporters associated with antigen-processing (TAP) into the ER, where residues of the N-extended sequence (if present) are trimmed by endoplasmic reticulum aminopeptidase proteins (ERAPs). Subsequently, the mature epitopes are loaded onto HLA-A*02:01 and enter the ER–Golgi transport pathway. After exocytosis, CMV peptide–HLA-I complexes are presented on the surface of cancer cells, which can then be recognized and eliminated by anti-CMV CD8^pos^ T cells (Figure 1).

In their study, Jung et al. injected TEDbodies and anti-CMV CD8^pos^ T cells into NSG mice bearing pre-established orthotopic MDA-MB-231 xenografts or subcutaneous HCT116 colorectal cancers. Treatment of mice with TEDbodies reduced tumor volumes by 40% and 70%, respectively, and increased numbers of tumor-infiltrating anti-CMV CD8^pos^ T cells by 15-fold compared to controls. Additionally, TEDbody treatment was combined with the agonistic OX40 antibody or the antagonistic PD-1 antibody pembrolizumab. Compared with monotherapy, co-administration of TEDbodies and agonistic OX40 antibody reduced the tumor volume by ~45% and increased the number of tumor-infiltrating anti-CMV CD8^pos^ T cells. Intriguingly, despite cell surface expression of PD-1, pembrolizumab did not enhance the efficacy of TEDbody treatment.

TEDbodies smartly take advantage of the natural antigen-processing and -presentation machinery of cells to mimic CMV infection by presenting CMV peptide–HLA-I complexes on the surface of cancer cells. However, to evade immune recognition, both antigen-processing and -presentation are frequently modified or defective in cancer cells [40]. As TEDbodies fully rely on an intact antigen-presentation machinery, down-modulation of or defects herein will likely render cancer cells insusceptible to this form of therapy.

#### 4.1.2. APECs

**Figure 2:**
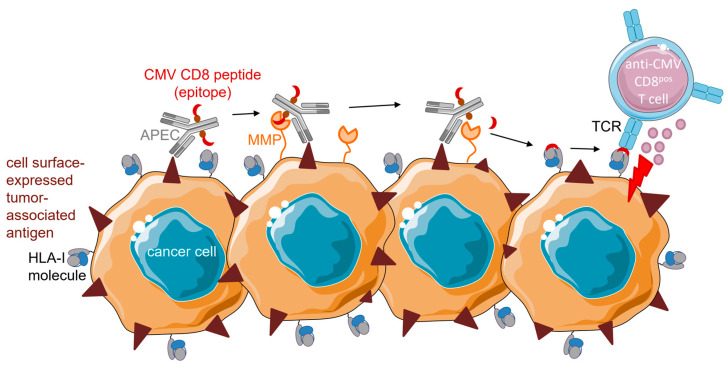
Proposed mode of action of APECs (adapted from Millar et al. [41]). APECs deliver HLA-I-restricted CMV peptide epitopes to the surface of cancer cells, thereby rendering them susceptible to elimination by pre-existing (inflationary) anti-CMV CD8^pos^ T cells, as follows: tumor-directed antibodies bind to corresponding target antigens on cancer cells. Cancer-associated matrix metalloproteases (MMPs) cleave the linker used to conjugate the CMV peptide of choice to an antibody moiety. Subsequently, the CMV peptide is released and binds to an ‘empty’ HLA-I molecule present on the surface of cancer cells. Anti-CMV CD8^pos^ T cells can now recognize and eliminate CMV peptide-presenting cancer cells.

Millar et al. (2020) developed an alternative strategy which circumvents the need for intracellular CMV peptide processing and loading. Antibody–peptide epitope conjugates (APECs) consist of CMV-derived peptide epitopes conjugated to tumor-directed antibodies via a linker [41]. This linker was designed to be susceptible to cleavage by cancer-associated matrix metalloproteases (MMPs). When APECs bind to the corresponding target antigen on cancer cells this linker is cleaved, after which the CMV peptide epitope is released. Subsequently, the peptide epitope is taken up and presented by an empty HLA-I molecule on the surface of cancer cells. Consequently, these CMV-‘redecorated’ cancer cells are recognizable for elimination by cognate anti-CMV CD8^pos^ T cells (Figure 2). In normal cells, cancer-associated MMPs are essentially absent so that APECs remain intact and the CMV peptide epitope will not become available for HLA-I presentation.

Using a library of 96 APECs containing short protease recognition sequences for cancer-associated proteases, MMP2 and MMP14 were identified to have the most suitable protease sites for generating immunogenic CMV peptide epitopes. The HLA-A*02:01-restricted CMV pp65 peptide epitope NLV was predominantly used in APECs, but HLA-A*01:01- and HLA-B*08:01-restricted CMV peptide epitopes were also found to be functional. The tumor-directed domains of APECs were based on the clinically approved antibodies cetuximab (anti-EGFR) and rituximab (anti-CD20). MMP14–cetuximab APEC was evaluated in NOD/SCID mice xenografted with breast (MDA-MB-231), liver (SNU-475), or lung cancer cells (MGH-1088). Mice were reconstituted by adoptive transfer of human anti-CMV CD8^pos^ T cells. In the breast cancer mouse model, MMP14–cetuximab APEC-treated mice showed a ~60% reduction in tumor volume compared to the control groups. Median survival in the breast and liver cancer mouse models was prolonged by 14 and 6 days, respectively. In the lung cancer mouse model, MMP14–cetuximab APEC-treatment did not prolong median survival, while overall survival was significantly improved in the intervention group despite the presence of right censoring. The study was terminated 44 days after the control group reached 0% survival. At this point, the survival rate of the intervention group was still 50%.

This proof-of-concept study was followed up by Zhang et al. (2022), who established an experimental pipeline to create patient-specific APECs for ovarian carcinoma treatment [42]. APECs were customized by selecting highly expressed targets, modifying protease cleavage sites to be patient- and tumor-specific, and utilizing viral antigens that elicit patient-specific T cell responses. Most patients in their cohort were suitable for MMP7 and the HLA-A*02:01 CMV pp65peptide epitope NLV. EpCAM-directed APEC, coined EpCAM-MMP7-CMV APEC, was designed because EpCAM was (over)expressed in 95% of their patient-derived ovarian carcinoma samples. In addition to CMV, EBV and Influenza antigens were evaluated, but the frequency and prominence of the antigen responses were inferior to those of the CMV epitopes. In vitro EpCAM-MMP7-CMV APEC bound to primary ascites-derived ovarian carcinoma cells and stimulated NLV-specific T cells. In vivo studies in immunocompromised zebrafish confirmed that EpCAM-MMP7-CMV APEC recruits anti-CMV_pp65_ CD8^pos^ T cells into the tumor mass and conveys robust cancer cell elimination. Additionally, a xenograft tumor model using NSG mice showed a ~50% decrease in tumor burden and 23 days prolonged median survival in animals treated with EpCAM-MMP7-CMV APEC.

In contrast to TEDbodies, APECs bind directly to empty HLA-I complexes on the surface of cancer cells and hereby circumvent potential defects in the intracellular antigen-processing and -presentation machinery. However, APECs rely on the presence of empty HLA-I complexes on the surface of cancer cells. Unfortunately, in a subset of patients, cancer cells do not express (empty) HLA-I complexes [42]. Moreover, treatment with APECs in their current format is only possible if the cancer cells express suitable cancer-specific MMPs.

#### 4.1.3. Intratumoral (i.t.) Injection of CMV Peptide Epitopes

**Figure 3:**
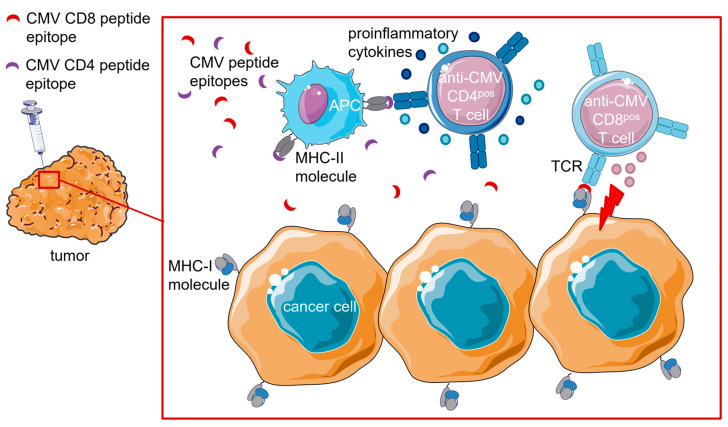
Proposed mode of action of intratumoral (i.t) injection of CMV peptide epitopes. I.t. injection delivers MHC-I-restricted and MHC-II-restricted CMV peptide epitopes into the tumor microenvironment. MHC-I-restricted CMV peptide epitopes are taken up by empty MHC-I molecules on the surface of cancer cells. Anti-CMV CD8^pos^ T cells can now recognize and eliminate CMV peptide-presenting cancer cells. MHC-II-restricted CMV peptide epitopes are taken up by empty MHC-II molecules on the surface of antigen-presenting cells (APCs). Anti-CMV CD4^pos^ T cells bind, become activated, and secrete proinflammatory cytokines that promote the induction of an adaptive immune response.

Another approach utilizing the presence of endogenous (empty) HLA/MHC complexes was examined by Çuburu et al. (2022) [43]. They opted to activate anti-CMV T cell responses locally by intratumoral (i.t.) injection of virus-derived peptide epitopes (Figure 3).

C57BL/6 mice were infected with MCMV. Of note, MCMV and HCMV infections are similar in terms of memory T cell inflation and broad T cell reactivity (also see Section 3). Six months post-infection, when memory inflation had been established and MCMV infection was in the latent phase, mice were subcutaneously injected with lung cancer cells (TC-1). These mice showed heavy infiltration of tumors by both inflationary and non-inflationary MCMV-specific CD8^pos^ T cells. Immune infiltration was even more prominent in tumors than in secondary lymphoid organs. A large fraction of tumor-infiltrating MCMV-specific CD8^pos^ T cells expressed CD69, indicating their differentiation into tumor-resident memory T cells.

Subsequently, mice were injected with MCMV-derived MHC-I (IE3, m38, and m45) and MHC-II (m139) peptide epitopes in the presence (or absence) of the innate immune modifier polyinosinic: polycytidylic acid (pI:C). Already in the absence of pI:C, MCMV-derived MHC-I peptide epitopes induced necrotic tissue formation, demonstrating the powerful cytotoxic function of tumor-infiltrating MCMV-specific CD8^pos^ T cells. Moreover, i.t. injection of virus-derived peptide epitopes induced immune infiltration of tumors, as demonstrated by the increased numbers of CD8^pos^- and Th1-T cells, including T cells that were not targeted by the injected peptides. Increased numbers of bystander NK cells, B cells, neutrophils, and macrophages were also observed in the tumor bed. Apparently, injection of MCMV-derived epitopes changed both the cellular composition and activation status of immune cells in the tumor microenvironment (TME). Injection of MHC-I peptide epitopes (without pI:C) reduced tumor growth by ~95% and prolonged median survival by 21 days. Remarkably, complete tumor regression was observed only in mice that received MCMV-derived MHC-II peptide epitopes alone or in combination with MHC-I peptide epitopes. Tumor-free mice were rechallenged with TC-1 cancer cells after 4 months and did not develop any palpable tumors (compared to age-matched naïve mice used as controls), suggesting that long-term antitumor immunity had been established.

Even treatment of immunologically ‘cold’ (B16-F10) melanoma xenografts with MCMV-derived MHC-I and MHC-II peptide epitopes reduced tumor volume by >80% and prolonged median survival by 18 days compared to the control group. Additionally, administration of MCMV-derived peptide epitopes caused significant activation of the TME, as was evident from a distinct increase in cancer-protective immune cells, particularly MHC-I peptide-specific CD8^pos^ T cells (>4-fold increase).

Finally, treatment with MCMV-derived peptide epitopes demonstrated high efficacy in a colon carcinoma model with a high mutation burden (MC-38 cancer cells xenografted into MCMV-infected C57BL/6 mice).

In conclusion, anti-MCMV CD8^pos^ T cells remain functional in both immunologically ‘hot’ and ‘cold’ murine tumor models and could overcome the immunosuppressive TME, although immune checkpoint inhibitors were not co-administered.

In contrast to TEDbodies and APECs, i.t. injection of CMV peptide epitopes involves MHC-II peptide epitopes and CD4^pos^ T cell responses. Simultaneous mobilization of antiviral CD4^pos^ and CD8^pos^ T cells appears to be favorable, as it leads to the production of multiple cytokines and chemokines, which activate several immune pathways and promote immune cell infiltration of tumors [43]. The practical application of this strategy remains to be determined, particularly for leukemia/lymphomas and tumors located in internal organs that are poorly accessible for direct injection. Moreover, i.t.-injected CMV peptides will be taken up by empty MHC/HLA on surrounding healthy cells as well. Hence, the severity of damage to normal tissues requires further evaluation, particularly because signs of systemic toxicity were observed in the study. Similar to TEDbodies, i.t. injection of antiviral epitopes does not rely on intact intracellular antigen-presentation pathways but requires the presence of endogenous empty HLA complexes on the cell surface.

### 4.2. Tumor-Directed Fusion Proteins Comprising CMV Peptide–HLA-I Complexes

**Figure 4:**
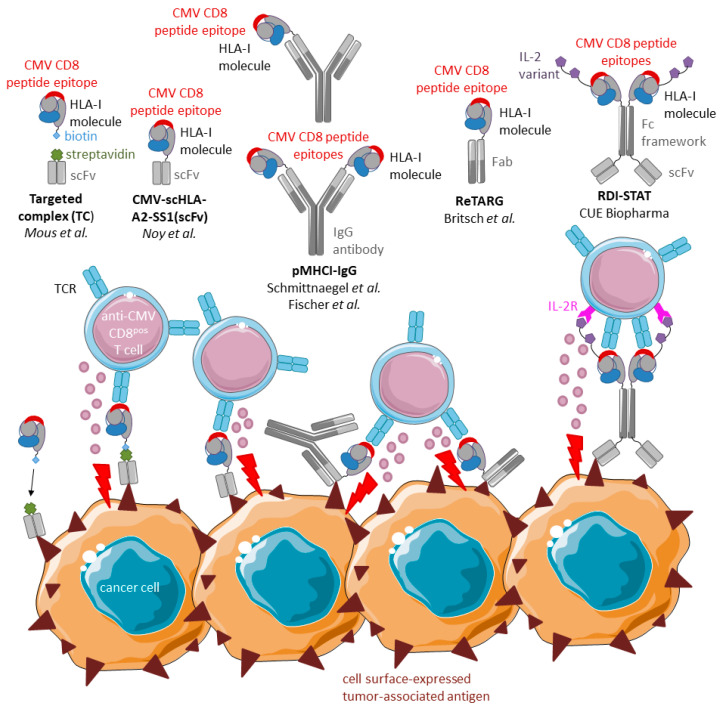
Schematic and proposed mode of action of various tumor-directed fusion proteins comprising CMV peptide–HLA-I complexes. Typically, tumor-directed fusion proteins comprising CMV peptide–HLA-I complexes contain a CMV peptide-equipped HLA-I/β2M complex and a tumor-directed antibody fragment (or whole antibody). These fusion proteins bind to the respective target antigen that is selectively (over)expressed on the surface of cancer cells via their antibody domain and thereby ‘present’ exogenous CMV peptide–HLA-I complexes. Anti-CMV CD8^pos^ T cells can then recognize and eliminate CMV peptide-presenting cancer cells.

To avoid the requirement for endogenous (empty) HLA-I complexes and bypass potential deficiencies in the antigen-presentation pathway, fusion proteins comprising CMV peptide–HLA-I complexes have been developed. These fusion proteins that redirect anti-CMV CD8^pos^ T cells towards cancer cells are equipped with a CMV peptide-loaded HLA-I/β2M complex fused to an antibody (fragment) moiety directed towards a tumor-associated cancer cell surface antigen (Figure 4).

Mous et al. (2006) redirected anti-CMV CD8^pos^ T cells to B-CLL cells [44]. They developed a targeted complex (TC), which consists of a streptavidin-tagged anti-CD20 scFv and biotinylated HLA-I molecules containing the HLA-A*02:01/HLA-B*07:02-restricted CMV pp65 peptide epitope NLV/TPR. When co-administered, anti-CD20 scFv binds to (CD20^pos^) B-CLL cells, after which it recruits the biotinylated CMV–HLA-I complex to the surface of cancer cells. TC-treated B-CLL cells were lysed in vitro by autologous anti-CMV CD8^pos^ T cells with an efficiency similar to that of peptide-loaded B-CLL cells. Moreover, anti-CMV CD8^pos^ T cells proliferated and produced proinflammatory cytokines (IFNγ, TNFα, and MIP-1β) in response to TC treatment.

Noy et al. (2015) constructed a recombinant molecule, designated CMV-scHLA-A2-SS1(scFv), by genetic fusion of the CMV pp65 peptide epitope NLV to the single-chain (sc)HLA-A*02:01 molecule and scFv anti-mesothelin antibody fragment SS1 [38]. In the presence of anti-CMV CD8^pos^ T cells, CMV-scHLA-A2-SS1(scFv) induced elimination of various cancer cell lines. Antigen-specific activation of anti-CMV CD8^pos^ T cells (CD25^pos^/ CD107^pos^) and cytokine secretion (IL-2 and IFNγ) occurred only in the presence of mesothelin-positive cancer cells. The in vivo efficacy was examined in BALB/c nude mice bearing human mesothelin-expressing N87 xenografts. Gastric carcinoma cells were subcutaneously injected into mice treated with CMV-scHLA-A2-SS1(scFv) in the presence of adoptively transferred human anti-CMV CD8^pos^ T cells. This treatment inhibited tumor growth by ~50% compared to the control group.

Schmittnaegel et al. (2015), generated fusion proteins, designated pMHCI-IgG, composed of a full tumor antigen-specific immunoglobulin fused to a single HLA-A*02:01 domain bearing the covalently linked CMV pp65 peptide epitope NLV [45]. pMHCI-IgGs were designed to target insulin-like growth factor 1 receptor (IGF-1) or melanoma-associated chondroitin sulfate (MCSP) on the surface of cancer cells. In the presence of anti-CMV CD8^pos^ T cells, pMHCI-IgGs facilitated the elimination of various tumor antigen-expressing cancer cell lines. Anti-CMV CD8^pos^ T cells from multiple healthy donors responded to pMHCI-IgG-loaded cancer cells without prior expansion. Importantly, the anticancer activity of anti-MCSP pMHCI-IgG was compared to an analogous anti-MCSP bispecific T cell engager (BiTE). Treatment of cancer cells with conventional BiTEs aims to activate and redirect all patients’ CD3^pos^ T cells towards cancer cells, irrespective of T cell subtype and/or intrinsic T cell receptor (TCR) specificity. Consequently, BiTE-based therapies are typically associated with massive secretion of proinflammatory cytokines, including IFNγ. High levels of proinflammatory cytokines may result in considerable in vivo toxicity, usually manifested as cytokine release syndrome [46]. Intriguingly, compared to anti-MCSP BiTE, anti-MCSP pMHCI-IgG showed comparable cytotoxic capacity but significantly reduced levels of T cell-secreted IFNγ, TNFα, and IL-2. Moreover, anti-MCSP pMHCI-IgG did not facilitate the production of cytokines that support Th2 and regulatory T cells. The in vivo efficacy of anti-MCSP-pMHCI-IgG was confirmed in MDA-MB-435 xenografted NOG mice adoptively transplanted with ex vivo-expanded human anti-CMV CD8^pos^ T cells and PBMCs. In this model, treatment reduced tumor volume and weight by ~40% and ~60%, respectively.

In a follow-up study, Schmittnaegel et al. (2016) aimed to identify the best-suited format of their fusion protein by comparing ‘single’ and ‘double’ pMHCI-IgGs [47]. Single pMHCI-IgGs carried only one pMHCI complex per IgG antibody, whereas double pMHCI-IgGs carried two pMHCI complexes, one fused to each variable domain of the antibody heavy chain. Single pMHCI-IgGs were less susceptible to non-specific activation. Double, but not single, pMHCI-IgGs induced TCR crosslinking without binding to target cells. Additionally, fusing pMHCI to the N-terminus of the variable domain of the antibody heavy chain reduced the intermembrane separation distance between T cells and cancer cells. An increase in intermembrane separation distance was previously shown to diminish the potency of T cell-mediated killing [48]. Hence, fusion protein formats that reduce the intermembrane separation distance appear to be more favorable.

Fischer et al. (2020) suggested that treatment with pMHCI-IgG complexes could also be applicable to CMV-seronegative patients with a matching HLA-I haplotype, if they would receive a vaccination against the CMV peptide contained in the particular pMHCI-IgG complex [49]. To investigate this in vivo, pMHCI-IgGs were engineered to contain an antibody sequence targeting murine fibroblast activation protein (mFAP), MCMV-derived peptide M38, and a murine MHC-I molecule. To trigger a robust peptide antigen-specific CD8^pos^ T cell response, immunocompetent C57BL/6N mice were vaccinated with a synthetic M38 peptide coupled to a DC-targeted XCR1 monoclonal antibody and received a booster injection with the peptide and cytokines. At the peak of the immune response, mice were i.v. injected with melanoma cells (B16-FAP) and pMHCI-IgGs were administered (i.v.) before or after. Pretreatment of mice with pMHCI-IgGs prevented tumor engraftment in the lungs, indicating that pMHCI-IgGs are effective in vivo when cancer cells are still in the blood pool. Delaying pMHCI-IgG treatment reduced tumor growth by ~60% but did not abolish tumor growth in the lungs. BiTEs and pMHCI-IgG were almost equally potent in eliminating melanoma cells. When using an advanced solid (MC38-FAP) colon carcinoma model, neither BiTEs nor pMHCI-IgGs protected mice from the tumor. The observed therapy resistance was not due to antigen loss, hampered penetration of pMHCI-IgG into the tumor mass, or lack of effector T cells recruited to the tumor. Both treatments triggered PD-L1 expression and increased the number of Tregs. Apparently, immune suppression mechanisms can easily shut off the functionality of CD8^pos^ T cells that have been retargeted to tumors by BiTEs or pMHCI-IgGs.

We recently investigated whether CMV peptide epitopes other than HLA-A*02:01-restricted CMV pp65 peptide epitope NLV, could elicit similar or even more potent antitumor responses. Previously, it was reported that individuals with both HLA-A*02:01 and HLA-B*07:02 alleles show dominance of anti-CMV CD8^pos^ T cell numbers specific for peptide TPR [5]. Additionally, HLA-B*07:02-restricted TPR-specific CD8^pos^ T cells preferentially expand compared to HLA-A*02:01-restricted NLV-specific CD8^pos^ T cells [50]. We designed EpCAM-ReTARG^pp65^, in which an HLA-matched CMV pp65 peptide, a single-chain soluble HLA-B*07:02/β2M molecule, and an EpCAM-directed Fab antibody fragment were fused in tandem [51]. We observed potent and specific elimination of various EpCAM-positive carcinoma cell lines as well as primary patient-derived cancer cells in the presence of HLA-B*07:02-restricted anti-CMV CD8^pos^ T cells. Comparison of EpCAM-ReTARG^pp65^ with the EpCAM-directed BiTE solitomab indicated a similar cancer cell elimination capacity but at ~55% decreased levels of T cell-secreted proinflammatory cytokines. Moreover, we reported for the first time a combinatorial treatment approach in which two fusion proteins recruiting anti-CMV CD8^pos^ T cells with distinct HLA/peptide-specificities were exploited. Combinatorial treatment with EpCAM-ReTARG^pp65^ and EGFR-ReTARG^IE−1^, which additionally recruits HLA-C*07:02-restricted anti-CMV_IE-1_ CD8^pos^ T cells, strongly potentiated selective cancer cell elimination compared to single-agent treatment, likely due to the concurrent cytolytic action of both cognate anti-CMV CD8^pos^ T cell clones.

CUE Biopharma developed Immuno-Selective Targeting and Alteration of T cells (Immuno-STATs), which were originally designed to prime and expand naïve and pre-existing anti-cancer T cell repertoires by co-delivering an engineered IL-2 variant (IL-2v) [52]. Immuno-STATs consist of a bivalent peptide–HLA-I complex and multivalent co-stimulatory molecules (affinity-attenuated IL-2v) built on an Fc framework. The HLA complex contains a tumor-directed peptide that engages TCRs in a tumor-selective manner. IL-2v was rationally designed to not bind IL-2 receptor (IL-2R) α, thereby largely preventing the IL-2-mediated activation and expansion of Tregs, which express high-affinity IL-2R. The interaction of T cells with IL-2v activates them in an antigen-presenting cell (APC)-independent manner. To prevent toxicity, the binding capacity of IL-2v to IL-2Rβ is mitigated.

Currently, an ongoing clinical trial is investigating Immuno-STATs to expand anti-HPV-1_6E7_ CD8^pos^ T cells (CUE-101) [53]. The expansion of anti-CMV CD8^pos^ T cells with Immuno-STAT was preliminarily examined using the CMV pp65 peptide epitope NLV presented on HLA-A*02:01. The interaction of CMV pp65 Immuno-STAT with the TCR of anti-CMV CD8^pos^ T cells in absence of co-stimulatory signals was sufficient to drive the proliferation of these cells in vitro and in a mouse model [54]. Recently, CUE Biopharma designed a bispecific redirected Immuno-STAT (RDI-STAT) molecule. RDI-STAT consists of a bivalent peptide–HLA-I complex and multivalent co-stimulatory molecules built on an Fc framework, which is fused to a tumor-directed antibody scFv moiety. This extended format of Immuno-STATs resembles other strategies described above. Compared to BiTEs, RDI-STATs prevented the production of toxicity-inducing levels of proinflammatory cytokines [55].

The introduction of IL-2v into fusion proteins comprising CMV peptide–HLA-I complexes could improve the capacity of anti-CMV CD8^pos^ T cells to eliminate cancer cells. Although IL-2v is affinity-attenuated, it may also activate T cells in the absence of cancer cells. Thus, possible in vivo toxicities must be thoroughly investigated.

In conclusion, several studies have underlined the functionality and efficacy of fusion proteins comprising CMV peptide–HLA-I complexes in recruiting inflationary anti-CMV CD8^pos^ T cells for cancer cell elimination. Targeted delivery of exogenous peptide-loaded HLA-I molecules renders these approaches independent of endogenous HLA expression levels in cancer cells. Several fusion protein formats have been evaluated, which consist of either one or two (streptavidin/biotin-coupled) parts and utilize whole IgG or only Fab/scFv antibody fragments., Multiple aspects must be considered when designing such molecules, including avidity, stability/in vivo half-life, and tissue-penetrating capacity. Usually, whole monoclonal antibodies have higher avidity than monovalent antibody fragments. High-molecular-weight protein drugs tend to be more stable and have a longer in vivo half-life but are limited in their capacity for extravasation and tissue penetration. Moreover, large CMV peptide–HLA-I-equipped fusion proteins may disturb the intermembrane separation distance of the immunological synaptic cleft and potentially diminish the potency of T cell-mediated killing. Despite their larger size, two-part fusion proteins utilizing streptavidin/biotin-coupling provide more flexibility to readily adjust them to fit patients with other HLA haplotypes [44]. A fusion protein format that balances all the above-mentioned criteria has yet to be developed. Further engineering to improve stability may help create the smallest molecule with the best shelf half-life. For example, linkers between the HLA-I heavy chain and β2M stabilize the structural integrity of the HLA-I complex. To stabilize the HLA-I peptide epitope in the binding groove, a linker between the peptide and the HLA-I heavy chain can be introduced. Artificial disulfide bonds between linkers and the HLA-I heavy chain can further improve stability [56].

## 5. Strategies Utilizing (Engineered) CMV T Cells in Adoptive Cell Therapy (ACT)

Expanded and reinfused anti-CMV T cells are commonly used to prevent CMV disease after hematopoietic stem cell transplantation (HSCT). Recently, anti-CMV T cells were evaluated in additional (new) forms of adoptive cell therapy (ACT). Chimeric antigen receptors (CARs) and TCRs were transduced into anti-CMV CD8^pos^ T cells to improve the in vivo persistence of CAR/TCR-engineered T cells. Moreover, anti-CMV T cells were activated ex vivo, expanded, and reinfused into glioblastoma patients to directly target CMV peptide epitopes expressed in a subset of glioblastomas.

### 5.1. CMV-CAR T Cells

Although remarkable treatment results have been achieved with CAR T cell therapy, lack of long-term persistence and poor expansion of transferred tumor-specific T cells remain major challenges [57,58]. (CAR) T cells require adequate co-stimulation to survive and proliferate. In some disease settings, particularly in patients with low or no disease burden, CAR stimulation (despite the improved functionality of newer generations of CARs) alone may be insufficient for T cell expansion [59]. Thus, repeated cycles of ex vivo expansion may be required to yield sufficient numbers of transferable CAR T cells. Unfortunately, prolonged ex vivo expansion potentially promotes T cell differentiation and exhaustion [60].

In contrast, antiviral T cells exhibit long-term persistence in cancer patients, which is caused by stimulation through their endogenous TCRs [61,62,63]. These observations have led to the development of strategies that express CARs in antiviral T cells to improve their in vivo persistence. In this way, CAR T cells receive co-stimulation following endogenous TCR interaction with latent virus antigens (cross-)presented by APCs. Improved in vivo persistence could reduce the required dose of transferred CAR T cells, which would in turn lower the risk of immune-related adverse events (irAE) [60]. Moreover, the use of antiviral CAR T cells may bypass the need for lymphodepleting chemotherapy, which is critical for the sufficient expansion of CD19-CAR T cells in patients with hematologic malignancies [64,65].

**Figure 5:**
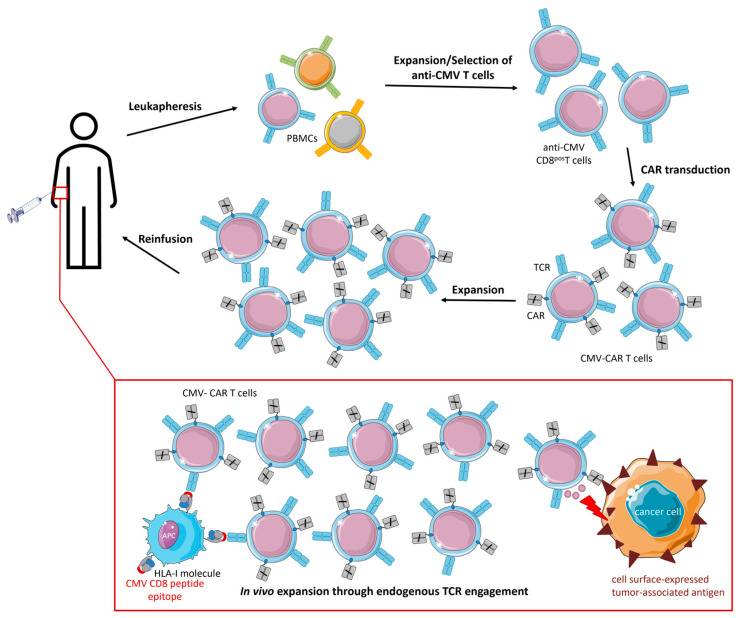
Therapeutic procedure and proposed mode of action of CMV-CAR T cells. PBMCs are isolated from cancer patient (or donor) blood using leukapheresis. Anti-CMV CD8^pos^ T cells are expanded by CMV peptide stimulation and subsequent supplementation with cytokines. Subsequently, anti-CMV CD8^pos^ T cells are transduced with CARs, expanded, and cryopreserved until intravenous reinfusion. CMV-CAR T cells expand (and are maintained) in vivo following endogenous TCR interaction with latent virus antigens (cross-)presented by APCs. Simultaneously, CMV-CAR T cells migrate to the tumor site and eliminate cancer cells via their TCR.

Utilizing inflationary anti-CMV CD8^pos^ T cells to produce CAR T cells ensures the availability of sufficient numbers of functional and potent effector cells with well-characterized favorable features, including low expression levels of PD-1. Various research groups have designed CMV-CAR T cells by isolating autologous or allogeneic anti-CMV CD8^pos^ T cells, expanding and transducing them with tumor-directed CARs (Figure 5). 

Ahmed et al. (2017) evaluated autologous CMV-Her2-CAR T cells in a Phase 1 dose-escalation study in patients with progressive Her2-positive glioblastoma [66]. Infusion of CMV-Her2-CAR T cells without prior lymphodepletion was well-tolerated without dose-limiting toxicities. Almost 50% of patients had clinical benefits, shown by a partial response with a reduction in tumor volume or stable disease. The transferred CAR T cells did not expand but were present in the peripheral blood for up to 1 year after infusion.

Cruz et al. (2013) generated allogeneic CMV-CD19-CAR T cells to treat patients with residual B cell malignancies after HSCT [67]. Allogeneic CMV-CD19-CAR T cells did not induce graft-versus-host disease (GvHD). However, the in vivo expansion and persistence of allogeneic CMV-CD19-CAR T cells remained suboptimal. In particular, appropriate CMV-CD19-CAR T cell engraftment was only observed in patients who were infused early post-transplant at a stage of lymphodepletion and high CMV load. The absence of high viral loads may result in insufficient co-stimulation to promote robust engraftment of CMV-CAR T cells. Early infusion may allow for better stimulation, increased expansion, and persistence of CMV-CAR T cells and facilitate more sustainable antitumor responses.

To promote the engraftment of CMV-CAR T cells, Caruana et al. (2015) created a whole-cell CMV vaccine to be administered to patients who were infused with CMV-GD2-CAR T cells [68]. The antitumor effect of CMV-CAR T cells was enhanced in vaccine-boosted compared to non-vaccinated mice as shown by a prolonged median survival of 19 days.

Wang et al. (2015) transferred CMV-CD19-CAR T cells into immunodeficient mice bearing human CD19-positive lymphomas [69]. The antitumor activity of CMV-CD19-CAR T cells was boosted by vaccination with CMV pp65 peptide. Vaccination also promoted CMV-CD19-CAR T cell expansion in vivo, thereby omitting the need for long-term ex vivo expansion. In a follow-up study, Wang et al. (2022) developed a large-scale clinical platform for generating CMV-CD19-CAR T cells [70]. The resulting CAR T cells were polyclonal and continuously expressed CD62L, CD27, and CD28, indicating engraftment and persistence of adoptively transferred T cells. Administration of a CMV vaccine ensured the maintenance of memory function of CMV-CD19-CAR T cells and improved their capacity to migrate to tumors. Intriguingly, compared to conventional CD19-CAR T cells derived from the same donor, CMV-CD19-CAR T cells appear to possess stronger effector functions against CD19-positive tumors. Currently, Wang et al. are initiating a clinical trial using clinical-grade CMV-CD19-CAR T cells in patients with intermediate/high-grade B cell non-Hodgkin’s lymphoma [70]. CMV-CD19-CAR T cells will be administered immediately after autologous hematopoietic cell transplantation. To facilitate the in vivo expansion of CAR T cells, patients will receive the CMV vaccine Triplex [71,72].

Taken together, CMV-CAR T cells demonstrated superior proliferation, survival, and antitumor activity in vivo compared to generic CAR T cells. It is well known that supraphysiological activation via CD3/CD28 drives T cell differentiation and exhaustion [73]. Replacing CD3/CD28 with viral antigen stimulation prior to CAR transduction apparently reduces CAR T cell differentiation and enhances the expression of homing molecules [70]. CAR T cell toxicities are often followed by prolonged B cell aplasia, which can trigger CMV infections [74,75]. Utilizing CMV-CD19-CAR T cells could simultaneously convey anti-CD19 effector functions while providing sufficient anti-CMV activity to prevent CMV infection [70]. Moreover, CMV-CAR T cells can be used preemptively after allogeneic HSCT to eliminate minimal residual disease and prevent CMV reactivation [69]. Remarkably, GvHD does not appear to be an issue when patients receive allogeneic antiviral T cells, even when they are derived from partially HLA-mismatched donors [76].

### 5.2. TCR-Engineered CMV T Cells

Given the improved survival capacity and antitumor activity of CMV-CAR T cells in vivo, it is not surprising that the use of anti-CMV CD8^pos^ T cells for other forms of ACT, such as TCR-engineered T cells, has also been investigated. Heemskerk et al. (2004) retrovirally transduced a leukemia-reactive TCR directed against minor histocompatibility antigen (mHag) HA-2 into anti-CMV CD8^pos^ T cells [77]. These TCR-engineered CMV T cells had dual specificity towards CMV and mHag HA-2 and showed similar TCR-specific cytolytic activity compared to generic TCR-engineered T cells. A follow-up study disclosed that repetitive stimulation skews TCR-engineered CMV T cells to predominantly express the triggered TCR [78]. Although the respective TCR expression levels are dynamic and can be reversed by stimulation of the less expressed TCR, that may not occur in particular in vivo settings. For example, in a minimal residual disease setting, TCR-engineered CMV T cell stimulation will mainly occur via viral antigens. Consequently, TCR-engineered CMV T cells will preferentially express the CMV-directed TCR and no longer react to antigens targeted by the engineered TCR. Interestingly, the expression threshold to induce proliferation and cytotoxic reactivity is higher for the artificially introduced TCR than for the endogenous TCR [78]. When applied in a (phase I) clinical trial, TCR-engineered CMV T cells were safely infused in five out of nine patients, but the overall efficacy of this treatment approach was too low to warrant further clinical development [79]. Only two patients had sufficient persistence and expansion of TCR-engineered CMV T cells. In the other three patients, TCR-engineered CMV T cells did not expand, probably because there was too little exposure of the antigen targeted by the artificially introduced TCR. Simultaneously, competition for membrane expression with the artificially introduced TCR also downregulated the expression of the endogenous virus-specific TCR. Consequently, mostly non-transduced antiviral T cells from the infused product expanded during viral reactivation after transplantation.

In conclusion, it appears to be more challenging to create TCR-engineered CMV T cells compared to CMV-CAR T cells, as forced expression of the artificial TCR downregulates the expression of the endogenous TCR. Additionally, artificial and endogenous TCR chains can pair, leading to the formation of mixed TCR complexes with undesired, possibly harmful specificities [77].

### 5.3. Direct Targeting of CMV Peptide Epitopes Expressed in Glioblastoma

An alternative approach for using anti-CMV T cells in cancer therapy is to directly target CMV peptide epitopes expressed in approximately 40% of glioblastoma multiforme (GBM) patients [80]. Intriguingly, these viral proteins are not expressed in surrounding normal brain tissue [81,82]. Targeting CMV protein-expressing GBM cancer cells may provide selectivity to eliminate them with no or only limited off-tumor toxicity towards normal cells of the central nervous system (CNS). Notably, in contrast to most humoral immune components, activated T cells pass through the blood-brain barrier.

Unfortunately, anti-CMV T cells present in GBM tumors appear to be incapable of eliciting effective antitumor responses. In particular, Crough et al. (2012) showed that the frequency of precursor anti-CMV CD8^pos^ T cells in GBM patients was similar to that observed in healthy CMV-seropositive individuals. However, terminally differentiated (CD27^neg^/CD57^pos^) anti-CMV CD8^pos^ T cells were more frequent in GBM patients [83]. The lack of expression of activation markers suggests a defect in the proliferative capacity of anti-CMV CD8^pos^ T cells in GBM patients [84]. Moreover, anti-CMV CD8^pos^ T cells isolated from resected GBM tumors lacked expression of CD103 and had augmented levels of the inhibitory receptors PD-1, TIM-3, and CTLA-4 [85]. In a substantial proportion of anti-CMV CD8^pos^ T cells (60–70%), the expression of TNFα, IFNγ, MIP-1b, and CD107a was impaired [83].

Apparently, in the TME of GBM, anti-CMV CD8^pos^ T cells are becoming exhausted and senescent, which is in sharp contrast to inflationary (effector–memory) anti-CMV CD8^pos^ T cells that can be found in other parts of the body of healthy individuals and cancer patients. Additional research is required to unravel why anti-CMV T cells in GBM patients are phenotypically distinct; however, the immunosuppressive nature of GBM is likely to be a contributing factor [86].

**Figure 6:**
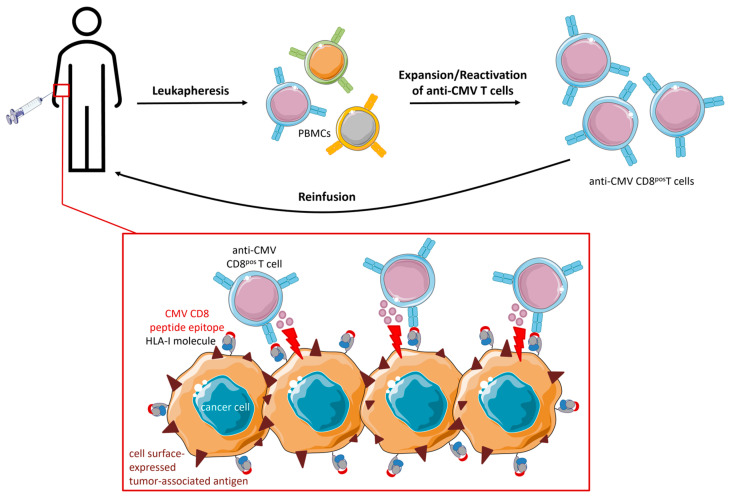
Therapeutic procedure and proposed mode of action of expanded/reactivated anti-CMV CD8^pos^ T cells for glioblastoma treatment. PBMCs are isolated from the blood of the patient using leukapheresis. Anti-CMV CD8^pos^ T cells are expanded by CMV peptide stimulation and subsequent supplementation with cytokines. A phenotypic analysis is performed to ensure adequate quality of anti-CMV CD8^pos^ T cells. After sufficient expansion, functional anti-CMV CD8^pos^ T cells are cryopreserved until intravenous reinfusion. Reinfused anti-CMV CD8^pos^ T cells migrate to the tumor site, recognize, and eliminate CMV-positive cancer cells.

Multiple clinical trials have been performed to determine whether expansion/in vitro stimulation and subsequent ACT could reconstitute anti-CMV CD8^pos^ T cell function in GBM patients. Phase I trials have assessed the treatment of either newly diagnosed or recurrent GBMs. ACT protocols vary across trials but basically consist of the following steps: harvesting of PBMCs from patients through leukapheresis, culturing in growth medium supplemented with HLA-I- and HLA-II-restricted peptide epitopes from CMV, and subsequent stimulation with recombinant cytokines, such as IL-2. The reinfused anti-CMV CD8^pos^ T cells are expected to migrate to the tumor site and recognize and eliminate CMV-positive cancer cells [86] (Figure 6). Optimal expansion protocols for anti-CMV ACT are still being developed.

Clinical (phase-I) trials showed that anti-CMV ACT enhanced progression-free survival (PFS) as well as overall survival (OS) and was associated with a reduced risk for side effects. The outcomes of these trials have been comprehensively reviewed by Sorkhabi et al. (2022) [86]. In short, Crough et al. (2012) showed that dysfunctional anti-CMV CD8^pos^ T cells in GBM patients could be ‘reinvigorated’ by ex vivo expansion [83]. Smith et al. (2020) reported that anti-CMV ACT improved OS by a median of 9 months, when it was initiated before recurrence of the tumor was evident. The median PFS of patients was 10 months [87]. Reap et al. (2018) assessed the efficacy of combining anti-CMV_pp65_ T cells from patients with GBM with autologous dendritic cells pulsed with CMV pp65-RNA and found that co-treatment enhanced the frequency of IFNγ^pos^, TNFα^pos^, and CCL3^pos^ polyfunctional anti-CMV CD8^pos^ T cells [88].

In conclusion, a subset of GBMs express CMV proteins, and cognate anti-CMV CD8^pos^ T cells are present in the TME. However, despite the correct antigen specificity, anti-CMV CD8^pos^ T cells appear to be dysfunctional in GBMs, a phenomenon not observed in other cancers and likely related to the immunosuppressive nature of this tumor type. Multiple clinical trials have shown that it is possible to restore the function of exhausted autologous anti-CMV CD8^pos^ T cells in vitro and reinfuse them into patients. Clinical benefits included enhanced PFS and OS.

## 6. Conclusions and Perspectives

Anti-CMV T cells are of particular interest for cancer (immuno)therapy because they are abundantly present, constantly renewable, and dominate the T-cell pool of the elderly, who are more often affected by cancer. In this review, we discuss various strategies for harnessing anti-CMV CD8^pos^ T cell responses for cancer (immuno)therapy. In short, several studies aimed to mimic CMV infection by (re)decorating malignant cells with viral antigens on endogenous or exogenous HLA-I complexes. APECs, TEDbodies, and i.t. injection of CMV peptide epitopes facilitate recognition by anti-CMV CD8^pos^ T cells by loading CMV peptides into HLA-I complexes expressed on cancer cells. All these strategies enhanced immune cell infiltration into the tumor, reduced tumor volumes, and prolonged survival in in vivo mouse models. Findings in tumor-directed fusion proteins comprising CMV peptide–HLA-I complexes include efficient cancer cell lysis, T cell activation and proliferation in vitro, and reduced tumor growth in vivo. Adoptive cell therapy (ACT) of engineered CMV T cells aimed to provide optimal co-stimulation and improved in vivo persistence for CAR/TCR-engineered T cells following endogenous TCR interactions with latent virus antigens. Indeed, CMV-CAR T cells demonstrated superior proliferation, survival, and antitumor activity in vivo compared to generic CAR T cells. However, attempts to design TCR-engineered CMV T cells have not been very successful thus far and the competition for surface expression between the endogenous and artificial TCR remains a major challenge. Ex vivo reactivation and ACT of dysfunctional anti-CMV CD8^pos^ T cells in GBM patients proved to be feasible and prolonged PFS and OS.

Taken together, a multitude of promising in vitro and in vivo data suggest that utilizing anti-CMV CD8^pos^ T cells to eliminate cancer cells could be an alternative next-generation approach in (immuno)therapy for both solid and non-solid cancers.

All treatment strategies utilizing anti-CMV CD8^pos^ T cells appear to have the following features in common:

Firstly, anti-CMV CD8^pos^ T cells do not require ex vivo manipulation or restimulation but display cytotoxicity directly after isolation from peripheral blood [45,51]. If expansion of anti-CMV CD8^pos^ T cells is necessary, for example due to insufficient T cell numbers, clinical-grade expansion protocols exist, and are safe and widely used. Obviously, ex vivo manipulation/expansion remains necessary for ACT-based strategies.

Secondly, the timing of the treatment appears to be crucial for obtaining optimal results. Treatment with CMV-CAR T cells worked better shortly after lymphodepletion, when the CMV viral load was high [67]. Administering reactivated anti-CMV CD8^pos^ T cells in GBM was more effective when initiated before recurrence of the tumor was evident [87]. Pretreatment with pMHCI-IgGs prevented tumor engraftment, while delayed treatment reduced but did not abolish tumor growth [49]. According to these findings, tumors should be as small as possible and the CMV viral load (and hence stimulation of anti-CMV CD8^pos^ T cells) as high as possible to facilitate optimal cancer cell elimination with anti-CMV CD8^pos^ T cells. To this end, pretreatment with cytoreductive agents could potentially improve the effectiveness of anti-CMV CD8^pos^ T cell-based strategies.

Thirdly, after initial cancer cell lysis by anti-CMV CD8^pos^ T cells, non-viral T cells targeting tumor-associated antigens are activated and appear to contribute to the anti-tumor response [43,87], a phenomenon known as ‘bystander effect’. Cancer cell lysis leads to increased cross-presentation of tumor antigens and hence neoantigen exposure/epitope spreading, which diversifies T cell responses [89,90]. Moreover, cancer cell lysis generates a proinflammatory TME, which recruits and activates other immune cells.

Fourthly, several studies have shown that anti-CMV CD8^pos^ T cell treatments can be boosted by vaccination [49,68,69,70]. Virus-derived antigens are likely to be shared among individuals and are not tumor type- and/or patient-specific. Therefore, anti-CMV vaccines are universally applicable [39]. Additionally, CMV vaccines could be used to render CMV-seronegative subjects susceptible to treatment strategies that rely on anti-CMV CD8^pos^ T cell responses [49]. However, CMV vaccination may not be able to (immediately) induce similarly high numbers of anti-CMV CD8^pos^ T cells in CMV-seronegative subjects as in CMV-seropositive subjects where they accumulated through memory T cell inflation.

Additionally, the following features seem to be shared among strategies that redirect inflationary anti-CMV CD8^pos^ T cells to attack cancer cells, such as TEDbodies, APECs, and fusion proteins comprising CMV peptide–HLA-I complexes (all discussed in Section 4):

Firstly, these strategies are relatively simple and broadly applicable, as characterization of individual mutations in cancer patients is not required [91].

Secondly, treatment approaches that selectively engage anti-CMV CD8^pos^ T cells reduce the levels of proinflammatory cytokines compared to conventional BiTE treatment [45,49,51], which is accompanied by the secretion of massive amounts of proinflammatory cytokines and known to be associated with serious irAEs. Significantly lower numbers of activated and cytokine-secreting T cells, achieved by selectively recruiting specific (CMV) T cell clones, could potentially improve in vivo tolerability.

Thirdly, the administration of drugs containing CMV peptide epitopes may promote the expansion of TME-resident anti-CMV CD8^pos^ T cells. Given that CMV antigens are highly expressed in some tumor types [92,93], the expansion of anti-CMV CD8^pos^ T cells in the TME is expected to be beneficial for long-term disease control.

Fourthly, small amounts of CMV-derived peptide epitopes presented on HLA-I are sufficient to activate anti-CMV CD8^pos^ T cells, likely due to the relatively high affinity of antiviral TCRs [43]. Interestingly, this does not necessarily seem to be the case for ACT-based strategies, as the absence of high viral loads appears to result in insufficient co-stimulation to promote CMV-CAR T cell engraftment. In TCR-engineered CMV T cells, competition for membrane expression with the artificially introduced TCR appears to downregulate the expression of the endogenous CMV-specific TCR. Nevertheless, the threshold to induce proliferation and cytotoxic activity was lower for the endogenous (CMV) TCR than for the artificially introduced TCR.

Of note, some of the strategies discussed here were also conducted utilizing other (inflationary) antiviral T cells against common viruses, such as EBV and Influenza. For example, EBV/influenza-CAR T cells [66,67,94,95] and fusion proteins containing EBV/influenza peptide-loaded HLA-I complexes [96,97,98,99,100,101] also exist but are beyond the scope of this review.

All strategies involving anti-CMV T cells naturally depend on the CMV infection status of patients. Approximately 83% of the general population is CMV-seropositive and is therefore eligible for anti-CMV T cell-based therapies [1]. Many strategies, except for those using expanded autologous anti-CMV CD8^pos^ T cells, require determination and adjustment of treatment to match the patients’ HLA-I haplotype. Multiple versions of APECs, TEDbodies, and fusion proteins comprising various CMV-specific peptides presented on corresponding common HLA haplotypes, such as HLA-A*02:01, HLA-C*07:02, and HLA-B*07:02, should be designed to make these strategies accessible to as many patients as possible. Given that CMV seroprevalence is highest in South America, Afrika, and Asia [2], HLA haplotypes that frequently occur in these populations are also of particular interest.

The evaluation of anti-tumor effects in immunocompromised in vivo models seems to be a major challenge, as injected anti-CMV CD8^pos^ T cells had insufficient capacity to survive and engraft in mice. Using NOG mice, which are not only lymphocyte-deficient but also lack functional macrophages, engraftment was improved [45]. However, co-administration of IL-2 or co-transfer of PBMCs was necessary to ensure sufficient survival of anti-CMV CD8^pos^ T cells. Similarly, in NSG mice, co-administration of IL15/IL-Rα-Fc was required to maintain anti-CMV CD8^pos^ T cell persistence [39]. In the future, better in vivo or alternative models to evaluate the potency of anti-CMV CD8^pos^ T cells for cancer (immuno)therapy need to be identified and/or designed.

As the surface expression of immune checkpoints limits T cell efficacy, combinatorial treatment approaches with immune checkpoint inhibitors, such as PD-1 blocking agents, should be more thoroughly investigated. PD-1 appears to have an inhibitory role in advanced solid cancer mouse models and can shut off functional anti-CMV CD8^pos^ T cells that are retargeted to tumors [49]. Circulating anti-CMV CD8^pos^ T cells responded to PD-1 treatment in patients with melanoma [102,103]. In contrast, co-administration of TEDbodies and pembrolizumab did not improve antitumor activity, although PD-1 and PD-L1 were expressed on anti-CMV CD8^pos^ T cells. Combinatorial treatment with TEDbodies and agonistic OX40 antibody had beneficial outcomes and warrants further investigations [39]. Recently, we discovered that cancer cells dynamically enhance CD47 cell surface expression, which coincided with acquiring resistance to pro-apoptotic effector T cell mechanisms [104]. Therefore, treatments combining anti-CMV CD8^pos^ T cell recruitment and CD47-blocking antibodies should be considered to improve their efficacy.

The diversity of approaches utilizing anti-CMV T cells for cancer (immuno)therapy may also open new avenues to improve outcomes by combining multiple strategies. For example, Immuno-STATs could be used to expand anti-CMV CD8^pos^ T cells in vitro before CAR transduction or reinfusion for GBM treatment. Moreover, CMV-CAR T cells and tumor-directed fusion proteins comprising CMV peptide–HLA-I complexes could be co-administered, thereby recruiting expanded and reinfused CMV-CAR T cells via their tumor-associated antigen (TAA)-targeting CARs as well as their TCRs to cancer cells. Thus, depending on the antibody (fragment) moiety of the fusion protein, multiple distinct TAAs could be targeted. Combinatorial treatment with CMV-CAR T cells and fusion proteins comprising CMV peptide–HLA-I complexes may be of particular interest for heterogeneous tumors and for treatments in which therapy resistance is acquired due to (CAR) target antigen loss in cancer cells.

In conclusion, the use of anti-CMV T cells for cancer (immuno)therapy has been rapidly growing, advancing, and diversifying in recent years. Although many aspects remain to be elucidated before anti-CMV T cell-based therapies can enter the clinical practice, these therapeutic approaches are highly promising, as they convey the natural potency of anti-CMV T cells to cancer cells and can be easily adapted for patient-tailored treatment.
